# A Recipe for Successful Metastasis: Transition and Migratory Modes of Ovarian Cancer Cells

**DOI:** 10.3390/cancers16040783

**Published:** 2024-02-15

**Authors:** Aleksandra Śliwa, Anna Szczerba, Paweł Piotr Pięta, Piotr Białas, Jakub Lorek, Ewa Nowak-Markwitz, Anna Jankowska

**Affiliations:** 1 Chair and Department of Cell Biology, Poznan University of Medical Sciences, Rokietnicka 5D, 60-806 Poznan, Poland; glodek@ump.edu.pl (A.Ś.);; 2 Gynecologic Oncology Department, Poznan University of Medical Sciences, 33 Polna Street, 60-101 Poznan, Poland

**Keywords:** ovarian cancer, metastasis, migration, CTC, cancer stem cells, transition modes, EMT, MET, EAT, AET, AMT, MAT

## Abstract

**Simple Summary:**

Ovarian cancer is one of the leading causes of cancer deaths among women with gynecological cancers. Most of the cases are diagnosed late, at an advanced stage when cancer cells spread to the surrounding tissues and to distant organs. Even though OC metastasis is the main cause of poor clinical outcomes, the understanding of the process remains elusive. This results in a lack of effective treatments for patients with advanced disease. This review focuses on the dissemination routes of OC as well as different transition modes adopted by tumor cells. These migratory modes increase the plasticity of cancer cells and the metastasis rate. Novel drugs, aimed specifically at the mechanisms responsible for the switch between different invasion modes, are promising approaches for OC treatment.

**Abstract:**

One of the characteristic features of ovarian cancer is its early dissemination. Metastasis and the invasiveness of ovarian cancer are strongly dependent on the phenotypical and molecular determinants of cancer cells. Invasive cancer cells, circulating tumor cells, and cancer stem cells, which are responsible for the metastatic process, may all undergo different modes of transition, giving rise to mesenchymal, amoeboid, and redifferentiated epithelial cells. Such variability is the result of the changing needs of cancer cells, which strive to survive and colonize new organs. This would not be possible if not for the variety of migration modes adopted by the transformed cells. The most common type of metastasis in ovarian cancer is dissemination through the transcoelomic route, but transitions in ovarian cancer cells contribute greatly to hematogenous and lymphatic dissemination. This review aims to outline the transition modes of ovarian cancer cells and discuss the migratory capabilities of those cells in light of the known ovarian cancer metastasis routes.

## 1. Introduction

The metastatic potential of cancer cells is dependent on many variables, but initially involves the cells gaining motility and acquiring a more aggressive phenotype. Such shifts in morphology and metabolism, which allow the cells to eventually migrate, invade, adapt to novel environmental conditions, and settle in new niches, are called cellular transitions. Several types of transition modes that change a cell’s phenotype have been reported. Two well-known modes, partially deciphered on the molecular level, are the epithelial-to-mesenchymal transition (EMT) and the mesenchymal-to-amoeboid transition (MAT) [1].

Some authors have distinguished other transition modes, including the mesenchymal-to-epithelial transition (MET), the amoeboid-to-mesenchymal transition (AMT), the epithelial-to-amoeboid transition (EAT), and the amoeboid-to-epithelial transition (AET). It is often difficult to speak of distinct categories, as the cells thrive in a labile state, exhibiting partial phenotypes of epithelial, mesenchymal, or amoeboid cells. Thus, transitions are signs of a perpetual cellular transformation that ensures successful cancer survival and spread.

Among all tumor types, ovarian cancer (OC) remains one of the most lethal, and this can be explained by the fact that more than 75% of women are diagnosed at later stages with a disseminated disease [2]. Although metastasis is one of the main causes of the deaths of OC patients, our understanding of this process is insufficient. Bearing the above-mentioned statistics in mind, it is important to look for answers in the process of OC transitions that create gateways for metastasis. Thus, this review aims to outline different transition modes of OC cells and discuss the migratory capabilities of those cells in light of the known OC metastasis routes.

## 2. Plasticity of Ovarian Cancer Cells

### 2.1. Epithelial-to-Mesenchymal Transition (EMT)

**Figure 1:**
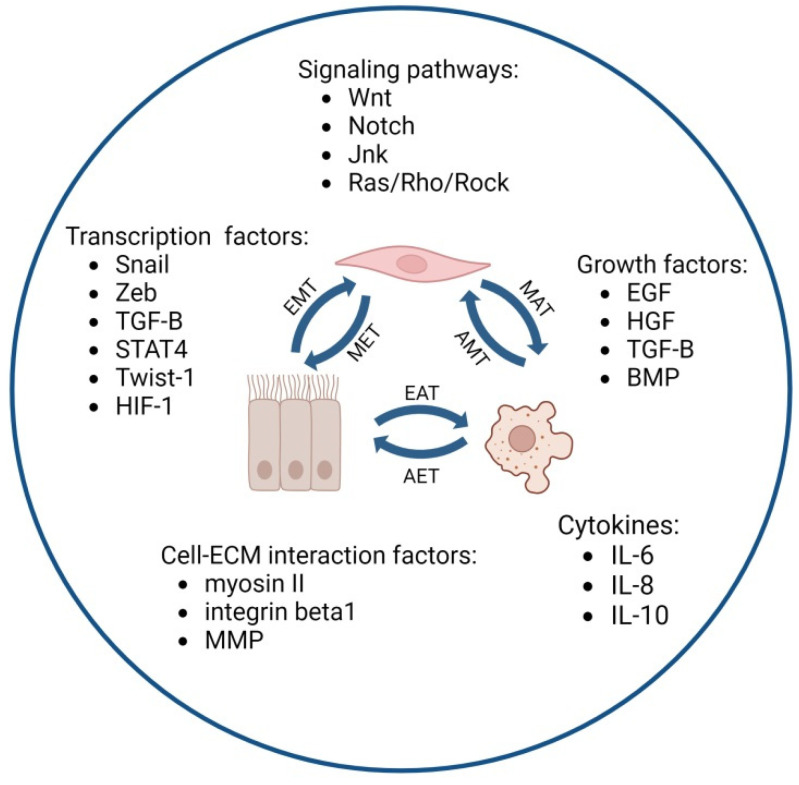
Factors associated with different transition modes of ovarian cancer cells (created with Biorender.com, accessed on 27 November 2023).

Considering the fact that most OC cells originate from epithelial cells, the type of transition that is most prone to occur is the epithelial-to-mesenchymal transition (Figure 1). This process includes a series of molecular changes that consequently lead to morphological, functional, and phenotypical alterations. A change in the phenotype from epithelial to mesenchymal is associated with the loss of cell polarity, cell–cell adhesion, and the gradual loss of attachment to the basement membrane via extracellular matrix (ECM) degradation. In turn, cancer cells gain a more spindle-like shape and an enhanced migratory capacity. An EMT allows OC to adapt to unfavorable conditions, including hypoxia or a nutrient deficiency, and promote chemoresistance as well as regulate the stemness of OC cells [3,4,5]. Once a cancer cell acquires a mesenchymal phenotype, it also becomes more motile and invasive, and after entering the ascites fluid, it may invade the neighboring tissues. OC cells usually invade the mesothelium and, less often, the peritoneum in a process called local or regional metastasis [6,7]. Nonetheless, the hematogenous and lymphatic routes of OC spread with distant metastasis are also common and, among other factors, require a high mobility, resistance to anoikis, and a high invasiveness of cells [8,9].

In OC, the induction of an epithelial-to-mesenchymal transition is believed to be mediated via the Wnt and Notch signaling pathways and persistent inflammation, in which cytokines, especially the interleukins IL-1, IL-6, IL-8, and IL-10, play a key role. An EMT is also supported by immune cells present in malignant ascites as well as oxidative stress and hypoxia at the site of the emerging tumor [10,11]. The latest research points to the significance of pyruvate dehydrogenase kinase 1, which regulates lactate production. The kinase promotes OC cell metastasis via α5β1 integrin and JNK/IL-8 signaling [12]. In addition, matrix metalloproteinases, growth factors (e.g., transforming growth factor beta—TGFβ, hepatocyte growth factor—HGF, and epidermal growth factor—EGF), bone morphogenetic proteins (BMPs) present in the ovarian neoplastic microenvironment, and regulatory proteins containing zinc finger structures (including Snail, ZEB, and Twist1) are believed to promote the EMT [11]. Recently, the STAT4 transcription factor has also been identified as a key player in the EMT and OC metastasis. STAT4 overexpression has been shown to be associated with poor outcomes for OC patients [13]. The EMT is considered the main factor promoting OC resistance to treatment. Both in vitro and in vivo studies have demonstrated that cancer cells that acquire a mesenchymal phenotype are resistant to carboplatin and/or paclitaxel, as well as to immunotherapy [3,9]. Thus, strategies blocking this process might make OC cells more susceptible to therapy and block metastasis. However, a partial EMT state of cancer cells has been reported to drive a build-up of ascites and peritoneal metastasis more effectively, compared to cells presenting with a full mesenchymal phenotype. Surprisingly, this transitional epithelial/mesenchymal (E/M) state seems to be linked with the highest aggressiveness of cancer cells. The spheroids found in ascites, which express both E-cadherin and N-cadherin, are more aggressive than pure epithelial multicellular aggregates [14]. Also, OC cells with an increased expression of ZEB1 in the transitional E/M state are very invasive, prone to migration, and resistant to anoikis. Finally, the ability of OC cells in a hybrid epithelial/mesenchymal state to drive tumor growth is linked with their characteristic features, which they share with cancer stem cells (CSCs). CSCs are a small group of cells within a tumor with capabilities of self-renewal that allow them to initiate tumors and drive metastasis and resistance to therapy [14].

### 2.2. Mesenchymal-to-Amoeboid Transition (MAT)

Under certain environmental conditions or due to therapeutical interventions, the cell adhesion or protease function of mesenchymal cells may undergo further changes and, as a consequence, the cells may gain amoeboid features. These features can include a shape deformation, the presence of bleb-like protrusions, a high contractility of actomyosin, a lack of cellular adhesion, weak cell–ECM interactions, and the proteolytic degradation of the surrounding matrix. The process of changing mesenchymal cells to amoeboid migrating cells is called the mesenchymal-to-amoeboid transition (Figure 1). The MAT involves rapid changes in the migratory mode and results in a round, but highly deformable, morphology and a boost in the stem cell potential, invasiveness, immunosuppression, and aggressiveness of cancer cells [15]. Studies conducted using OC spheroids have documented that such cell clusters may invade the mesothelium by displacing mesothelial cells from underneath the tumor. This process is based on the activation of nonmuscle myosin II and the generation of a traction force in an integrin- and talin-dependent manner [16].

The increased contractility of cells using amoeboid-like motility is promoted by the Rho/ROCK signaling pathway. This type of movement allows cells to squeeze through gaps in the extracellular matrix fibers and adapt to existing spaces or exert enough force to deform the ECM [15,17]. In OC, the Ras/Rho/ROCK/NF-κB signaling pathway induced by lysophosphatidic acid (LPA) can activate proteolytic enzyme secretion and, thus, promote cancer progression. Rho–ROCK signaling also rules out the tropism of metastatic OC cells in vitro [17]. Elevated RhoA levels correspond to OC progression, while an increased expression of RhoC is correlated with progression as well as a poor prognosis for patients. A recent study revealed that drug-resistant OC cells are more contractive and softer than drug-sensitive cells. In addition, cisplatin-resistant cells were demonstrated to employ mainly a mesenchymal mode of invasion. On the other hand, cells resistant to paclitaxel and those resistant to both of the mentioned therapeutical agents used an amoeboid mode of invasion. Both modes of invasion were regulated by the RhoA/ROCK2/myosin pathway [15,16,17,18]. Since the overexpression of Rho/Rock kinases has been linked to an increase in cancer cell dissemination and differences in the efficacy of the cisplatin action, the use of kinase-specific inhibitors to block cancer cell invasiveness may be applied in OC treatment [19,20]. In fact, the inhibition of RhoA, ROCK1, or ROCK2 by fasudil has already been reported to enhance cisplatin-induced growth inhibition and apoptosis in human OC cell lines via a reduction in HIF-1α expression [19]. The Rho/ROCK/myosin pathway mediates the gain of amoeboid features, but also of cancer-stem-cell-like properties. The stemness of cancer cells is supported via EMT gene regulation. Thus, the mesenchymal-to-amoeboid transition and the epithelial-to-mesenchymal transition are two modes of cancer cell invasiveness that are mutually interchangeable and that allow cells to adapt their mechanisms of migration to different environmental conditions [21,22].

### 2.3. Epithelial-to-Amoeboid Transition (EAT)

The mechanisms of the EMT and the MAT have been thoroughly studied in recent years, but the interplay between these transition modes remains elusive. Interestingly, some authors have reported the occurrence of a direct epithelial-to-amoeboid transition (EAT), called by others the collective-to-amoeboid transition (CAT) (Figure 1). Little is known, however, about the mechanisms behind this phenomenon. The number of studies documenting the EAT is still limited, and thus, the CAT or the EAT may be described using the results of single studies rather than summarized with an overall scheme [23,24]. Although there is no direct evidence that an EAT occurs in OC, it seems reasonable to expect that this process may be universal to many, if not all, types of cancer. Thus, in order to unveil this phenomenon, we present selected reports documenting the EAT in melanoma, hepatocellular carcinoma, breast cancer, and head and neck cancer.

The first studies documenting the CAT were published in 2002 and concerned migrating melanoma cells. The results of Hegerfeldt’s experiments revealed that the loss of adhesion due to the blocking of β1 integrin allowed cells to change their polarity and cohesion and caused cluster disruption [23]. As a consequence, individual cells detached from the cluster and migrated in a beta1-integrin-independent amoeboid manner. Another example of the CAT was documented in studies regarding the invasion of fibrosarcoma and breast cancer cells. Using an in vitro model, it was found that the separation of the anterior force-generating leading edge (containing β1 integrin, MT1-MMP, and F-actin) from a posterior proteolytic zone affected mechanotransduction and caused fiber breakdown and collagen remodeling [24].

More recent studies have reported the direct conversion from epithelial to amoeboid features of hepatocellular carcinoma in organoids and three-dimensional matrices, which mimic the physiological conditions. In such an environment, an EAT may be induced by the downregulation of NOX4 (an NADPH oxidase). NOX4 is essential for maintaining parenchymal structures, increasing cell–cell and cell–matrix adhesion, and impairing actomyosin contractility and amoeboid invasion. NOX4 gene deletions are common in HCC patients, and they are correlated with a higher tumor grade. The authors concluded that the loss of NOX4 in tumors is associated with an incline in actomyosin expression that promotes an EAT and eventually leads to a worse prognosis. Similar effects on the cytoskeleton may be achieved by silencing the epidermal growth factor receptor (EGFR) with a concomitant TGF-β treatment. Such a strategy induces an increase in the myosin II content in the cell cortex as well as the cell blebbing and amoeboid behavior of cancer cells. The blocking of EGFR and stimulation with TGF-β are associated with the upregulation of RhoC and Cdc42, two key regulators of amoeboid migration [25].

Lehmann et al. identified hypoxia as a factor that promotes an EAT. The deprivation of oxygen, such as the deprivation that occurs within a tumor mass, seems to promote a switch from collective migration towards amoeboid movement. Under normoxic conditions, cells migrate collectively, while hypoxic cells exhibit the downregulation of E-cadherin and disseminate individually from collective invasion strands. The matrix metalloproteinase (MMP)-independent migration of cells with a rounded morphology in an amoeboid manner lasted for hours, and even days, in 3D cultures of collectively invading breast cancer and head and neck cancer spheroids. This movement was mediated by an HIF-1-dependent mechanism. The hypoxia-induced EAT was related to the upregulation of the cysteine protease calpain-2, which limits β1 integrin activity and supports metastasis. Finally, the authors suggest that the observed amoeboid migration may be a common finale of different dissemination programs, dependent (Twist-mediated) and independent of an EMT [26,27]. A shift towards the amoeboid phenotype and such cell migration was also observed in cell lines in response to wall shear stress, a simulation of lymphatic-like flow. The biomechanical force, which drives this transition, triggers high levels of RhoA-ROCK signaling. Finally, adipokine factors were also shown to induce an EAT (as well as an EMT) in cancer cells [28,29].

To date, no such revelations have been reported for OC. This is not completely due to a lack of studies. Experiments using adipokine factors were conducted on OC, but these studies only confirmed the potential of cells to undergo an EMT with no signs of an EAT [29]. Still, the number of studies referring to a CAT/EAT in OC remains limited, and thus, it is difficult to say whether this phenomenon is exclusive to OC [30].

### 2.4. Redifferentiation of Cells

The process of metastasis requires multiple events of an EMT, but also requires the reverse process of a mesenchymal-to-epithelial transition (MET), which allows secondary tumor formation (Figure 1). The MET includes the conversion of motile, multipolar mesenchymal cells to polarized epithelial cells. Yet, the mechanisms driving the MET are not as clear as those underlying the EMT. Consequently, since many cancer cells are found in an intermediate or partial state of transition, the EMT and the MET may not be considered binary systems. Much of our understanding of the mechanisms that control the MET comes from studies on embryogenesis and cultured epithelial cells [31].

The typical re-epithelialization process involves the polarization of the cells that generate the apical membrane and the basolateral membrane, which guarantees adhesion to the extracellular matrix. The connection with the ECM is established through the transmembrane protein nectin, which forms a scaffold and helps recruit the actin-binding protein afadin and E-Cad. In turn, E-cadherin interacts with β-catenin, p120 catenin, and α-catenin, which ensures a linkage of the complex with the actin cytoskeleton. This evokes a signaling cascade by indirectly recruiting Rac and Rho. Eventually, this triggers cortical actin network formation and reinforces the adherens junction [30,31,32,33,34,35].

During carcinogenesis, an MET was shown to be induced by several different factors, such as the c-met protooncogene, 5-azacytidine, a DNA methyltransferase inhibitor, the transcription factor Snail, and the loss of Frizzled-7, which encodes a receptor for Wnt signaling [36]. The mechanism behind the MET is believed to also be triggered and regulated by environmental factors and dependent on reversible epigenetic modifications. Intriguingly, studies on OC cell lines have demonstrated that the mesenchymal-to-epithelial transition may be a result of the overexpression of miR-429, a member of the miR-200 family of microRNAs. This process was also dependent on the decline of ZEB1/2 expression, which are said to be EMT inducers. Thus, the interplay between the ZEB and miR-200 families seems to be a key factor regulating epithelial differentiation in OC [37,38].

OC cells found in the MET state are indeed characterized by gradual dedifferentiation with an increased E-cadherin expression, on a level similar to or lower than that of the primary tumor cells. This phenomenon is characteristic of the transcoelomic route of dissemination and allows cells to develop macroscopic peritoneal and omental metastases [39]. Studies on metastatic cancers have suggested that the invasive behavior of cancer, including OC, may also be linked with the amoeboid-to-epithelial transition and the reestablishment of E-cadherin-dependent adherens junctions. Such a process is induced by the PDGF, and Rho kinase inhibitors may be used to block invasive cancer cells [39,40]. Still, as documented by many authors, cancer cells may present with a dynamic overlap of the EMT and the MET, the MAT and the AMT, and the EAT and the AET rather than discrete epithelial, mesenchymal, or amoeboid behavior. In fact, many tumor tissues are characterized by the presence of subpopulations of cells that demonstrate different penetrations of epithelial and mesenchymal characteristics, including cells in a hybrid state. Typical of this state, and also described as a partial EMT (pEMT), is the different cellular plasticity associated with an increased metastatic potential. In this context, transitions may be considered a continuum of states that satisfies the growing needs of the cells to survive, invade, and colonize/establish new lesions [40,41].

## 3. Migratory Modes of Ovarian Cancer Cells

Cellular transitions change cells so that they acquire new favorable phenotypes. Depending on the environment and needs, cells undergo an EMT, MAT, or EAT, which not only affects the morphology and metabolism of cells, but also their motility. Cell migration is a well-known process that occurs during embryonic development, wound healing, and immunological surveillance. In pathologies such as cancer, migration is responsible for the dissemination of tumor cells [31,38,42].

**Figure 2:**
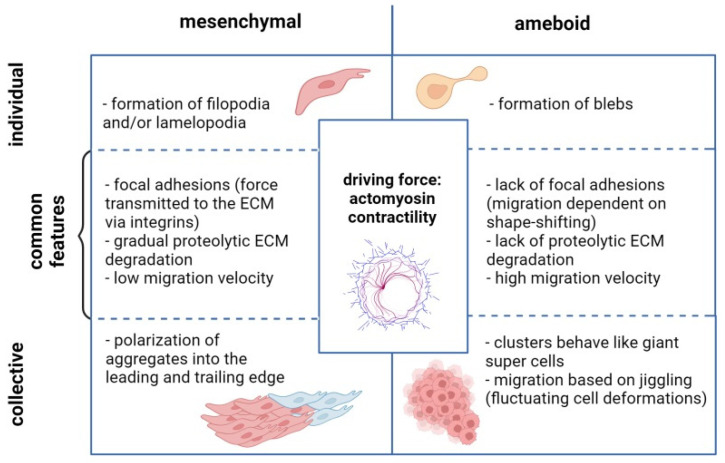
Characteristic features of different migratory modes of ovarian cancer cells (created with Biorender.com, accessed on 27 November 2023).

The spread from the primary tumor mass is typically facilitated via four main migratory modes: (1) migration as individual cells displaying a mesenchymal phenotype; (2) the migration of single cells displaying an amoeboid phenotype; (3) the dissemination of mesenchymal cells in clusters—collective (mesenchymal) cell migration; and (4) the dissemination of clusters of amoeboid cells—collective amoeboid cell migration (Figure 2) [25,27,43]. Although these migratory strategies seem very different, the fact is that the mechanisms of force generation and transmission modes are uncommonly conserved among cells of different origins. Within this conserved model, the shape and locomotion mode are determined by the degree of adhesion of the cells to the ECM and the actin and myosin activity. These factors, which adapt to intrinsic (expressed genes, signaling pathways) and extrinsic (chemical, mechanical) stimuli, determine the dynamics and the mode of migration of cells [44].

### 3.1. Migration of Individual OC Cells

The migration of individual cancer cells that have undergone a complete EMT utilizes the cytoskeletal (actomyosin) contractility. This mode is based on the clutch model, in which cells anchored in the ECM exert forces on the matrix by contracting the actomyosin cytoskeleton. The force is then transmitted from the cytoskeleton to the ECM via integrins and the adaptor proteins that link the integrins to actin. The turnover of these focal adhesions generates the pulling force, which moves the cells forward [42]. Mesenchymal motility is facilitated by the formation of filopodia and/or lamellipodia at the front of the cell, in which actin filaments push the plasma membrane, thereby contributing to the translocation of the cell. The degradation of the ECM by matrix metalloproteinases and other proteases is also required for mesenchymal cells. The proteolytic digestion of the ECM molecules generates routes for the migrating cancer cells. However, since the formation of focal contacts and their turnover is rather slow, the velocity of this migration mode is rather unspectacular [45].

In contrast to mesenchymal migration, amoeboid movement is independent of integrins and the proteolytic degradation of the ECM by metalloproteinases. The effective infiltration of the narrow gaps of the surrounding ECM is possible thanks to the rapid deformation of the cells, which squeeze in between the ECM fibers. Amoeboid cells are true shapeshifters that reorganize their cortical actin cytoskeleton, elongate, and contract in high-speed cycles, all thanks to increased actomyosin contractility driven by the activation of Rho/ROCK signaling. The amoeboid motility of cells is mediated by different types of Rho GTPases, including RhoA, RhoB, and RhoC OC [15,19,46,47].

The penetration of the ECM by amoeboid cells seems to also be aided by spherical membrane bulges called blebs, actin-rich pseudopodia, and/or highly contractile uropods [48]. Blebs are cell protrusions that arise due to myosin II-driven changes in the intercellular hydrostatic pressure, and they are said to act as mechanotransduction mediators and propel movement [49]. The fact that amoeboid cells do not establish intercellular adhesions and, in general, exhibit weak cell–ECM interactions allows them to move with a relatively high velocity (2–30 mm/min) [42]. Taken together, this ranks amoeboid migration as primitive, but highly efficient, which guarantees an escape route from densely packed tissue [50].

As mentioned earlier, the amoeboid movement requires a high level of Rho GTPase activity to drive actomyosin contractility. In fact, in epithelial OC, spheroids believed to act as metastatic units employ a myosin-generated force to breach the mesothelium of peritoneal organs and attach to the submesothelial connective tissue. The results of in vitro studies have demonstrated that the cell morphology, focal adhesion, cytoskeletal organization, and actomyosin contractility of OC SKOV-3 cells are positively correlated with substrate stiffness, which, in turn, enhances EOC cell migration [51].

Since it has been confirmed that amoeboid movement facilitates local tissue invasion in vitro, it is very plausible that this migration mode contributes to metastatic disease progression. Nonetheless, the significance of amoeboid motility in driving metastasis (especially in terms of intravasation into blood and lymphatic vessels) remains elusive [52].

### 3.2. Collective Migration of OC Cells

Another type of migration mode is associated with the movement of multicellular units with preserved cell–cell junctions. This migratory mode, known as collective (mesenchymal) cell migration and called tumor budding by some authors, has been observed in histological specimens of human cancers and mice during spheroid xenograft transplantation [53,54].

Mesenchymal cells found in clusters operate hierarchically. The polarization of the cells leads to the formation of a “leading edge” and a “trailing edge”. Different function allocation requires a change in the expression of the selected genes. Nonetheless, coordinated communication within the multicellular units guarantees the integration of mechano-sensing and the smooth transmission of traction forces [53,54,55]. Similarly to the EMT process, the motility of these cells, especially leading cancer-associated fibroblasts (CAFs), is dependent on actin contractility, the ECM–cell adhesion mediated by integrins, and the partial proteolytic degradation of the ECM. In this way, collective cell migration, analyzed at the cellular level, is similar to mesenchymal motility. Still, since cells migrating collectively in this mode demonstrate epithelial properties, their phenotype is often described as hybrid epithelial/mesenchymal (E/M) or a partial EMT, as they are positive for both E-cadherin and N-cadherin [54,56]. According to research data, the partial EMT state of OC cells is a more aggressive phenotype than that of cells after complete transition, and it promotes ascites formation and peritoneal metastasis. Consequently, OC cells in a hybrid E/M state display stem cell features with a greater capacity for self-renewal, are more resistant to anoikis, and may drive tumor growth [43,57].

A recent study documents that cell clusters obtained from patients’ explants may exhibit collective amoeboid migration. The premises of such a migration type were seen in histological specimens in the form of tumor spheres with an inverted polarity. This hypothesis was confirmed using a model, in which cancer cell aggregates were cultured in nonadhesive microchannels. Under such conditions, the cell clusters behaved like giant super-cells and migrated without focal adhesions. The most profound motility was observed for stomach and uterus tumor explants, but also, to a limited extent, in the case of OC cells. The results of the experiment illustrate that collective amoeboid movement relies on polarized actomyosin contractility and fluctuating cell deformations (jiggling). The authors thus propose that the model that best describes the behavior of these cells is the actively polarized jiggling elastic solid model [58].

An interesting form of migration, observed again for both mesenchymal and amoeboid cells, is multicellular streaming, in which cells follow one another in a strand. On the other hand, solid strands are cells protruding directly from the main tumor mass. Also, based on the analysis of histological specimens, other authors have distinguished collective motility in the form of sprouts, strands, clusters, and luminal or hollow structures [59].

## 4. Metastatic Routes in Ovarian Cancer

### 4.1. Transcoelomic Metastasis

**Figure 3:**
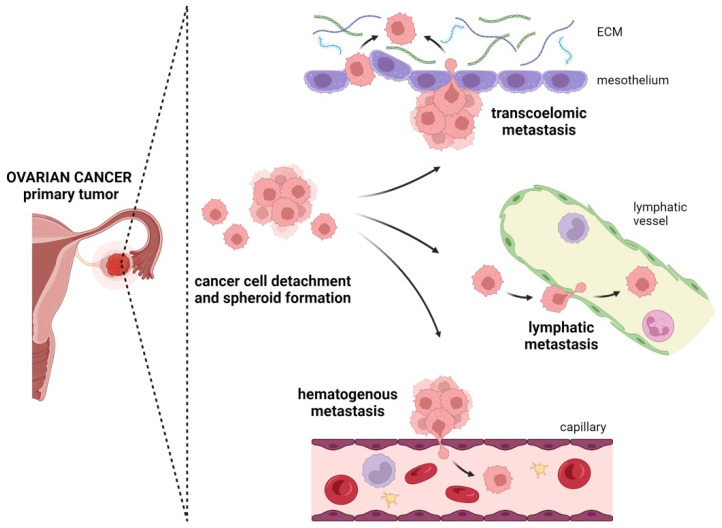
Metastatic routes of ovarian cancer (created with Biorender.com, accessed on 27 November 2023).

The most common method of OC cell dissemination is the transcoelomic route [15,16]. In this type of metastasis, single cancer cells, multicellular aggregates, and spheroids seed into the mesothelial layer and organs of the peritoneal cavity (Figure 3) [16,17,60].

The peritoneal cavity is lined with a single layer of cells called the mesothelium and is characterized by the production of high-molecular-weight hyaluronan (HA). In the early stages of cancer, the presence of this type of HA prevents the adhesion of malignant cells present in the peritoneal fluid. However, along with the progression of cancer, certain proinflammatory conditions are created in the peritoneal cavity, and the metabolism of the mesothelial cells changes. This results in the production of low-molecular-weight HA. The change in the extracellular matrix composition makes the mesothelium more permeable and enables the anchorage of tumor cells and spheroids [61].

Cancer cells adhere to the peritoneal lining thanks to the direct interactions of a transmembrane glycoprotein—CD44—with the aforementioned hyaluronic acid [62,63]. Aggregates of adherent cells of over 50 μm in diameter require the presence of a secreted proteoglycan named versican. Still, the expression of CD44 promotes spheroid formation, while versican facilitates the interaction between CD44 and hyaluronan and, thus, regulates the peritoneal adhesion and spread of cancer spheroids [64]. Interestingly, the hyaluronan–CD44 axis induces the epithelial-to-mesenchymal transition—EMT—process and affects cancer cell stemness, probably through Akt pathway signaling [65].

The research on OC adhesion also points to the significant role of mesothelin. Mesothelin is overexpressed in primary cancer and matched peritoneal metastasis. Its presence on the surface of mesothelial cells allows for the anchorage of tumor cells through the interaction with the ovarian antigen CA125 (MUC16). Mesothelin promotes cell survival in suspensions as well as invasion through the mesothelium and the eventual spread within the peritoneal cavity [66]. Other molecular players that support the process of OC cell adhesion include L1 adhesion molecule (L1CAM), a chemokine receptor fractalkine (CX3CR1), α_5_β_1_ integrins, and CD24. These agents interact with their partners present on the surface of the mesothelium, namely neuropilin-1, CX3CL1, fibronectin, and P-selectin, respectively [67,68,69,70]. The successful invasion of metastasizing cells requires the breaching of the mesothelial monolayer. This process is aided by certain enzymes overexpressed by OC cells. Adherent cells are known to utilize alcohol dehydrogenase 1B (ADH1B), prominin-1 (PROM1), and N-cadherin, with the last also being used by spheroids. Interestingly, cancer cell aggregates can disrupt the mesothelial lining with myosin-generated traction force [71,72].

After the clearance of the mesothelium, cancer cells invade the submesothelial parenchymal tissues with the help of a transmembrane collagenase termed membrane type 1 matrix metalloproteinase (MT1-MMP) [73]. The high expression of this enzyme is correlated with the increased invasion of epithelial OC cells [74]. Lengyel and coworkers reported that the attachment of OC cells to the peritoneum is also aided by the proteolytic activity of matrix metalloproteinase 2 (MMP-2), which cleaves the extracellular matrix (ECM) proteins fibronectin (FN) and vitronectin (Vn). The cancer cells bind to the FN and Vn fragments as well as their receptors, α_5_β_1_ and α_5_β_3_ integrin, and thus, they attach to the peritoneal surfaces [75].

Research on the omentum has revealed that OC cells exhibit a specific metastatic tropism towards this adipose-rich tissue. The greater omentum contains a network of blood vessels and energy reserves in the form of adipose tissue, which provides favorable conditions for tumor development. In addition, the adipose tissue of the omentum creates pockets that are not covered with mesothelium, which facilitates the accumulation of cancer cells. Indeed, research conducted using a mouse model verified that OC is more likely to metastasize into the mesentery and the greater omentum, which are fat structures coated with mesothelium rather than located directly in the intestinal wall. Tumors attached to the intestine encounter restrictive barriers and, thus, grow into the intraperitoneal space [76,77].

For this reason, a total omentectomy is considered essential to debulking surgery, which is the mainstay procedure in OC patient treatment, especially in the case of metastatic OC. An aggressive surgical cytoreduction and the removal of all tumors extends the lives of ovarian cancer patients [78]. As shown by an exploratory analysis, an omentectomy improved the 5-year cause-specific survival for stage II–IIIA OC patients [60,79,80,81,82]. However, the complete removal of a grossly normal omentum does not always translate into clinical benefits for the OC patient. An analysis of OC patients with a cancer stage below IIIA indicated that the removal of the omentum did not improve the survival rates [83]. Thus, randomized controlled trials are needed to fully address this problem.

The most abundant and important cellular component of both omental and peritoneal tissue is adipocytes [84]. Adipocytes assist in many biological functions, such as cellular metabolism, inflammation, and cancer development [85]. The interactions between adipocytes and cancer cells changes adipocytes into “cancer-associated adipocytes”. Activated adipocytes secret large amounts of lipids, adipokines, tumor-promoting factors, and hormones, which induce metabolic changes in both cancer cells and adipocytes [86]. Human omental adipocytes may also promote the homing, migration, and invasion of cancer cells. They contribute to tumor growth and progression by enabling the generation of energy. This can be achieved by the direct transfer of lipids and the secretion of adipokines, interleukins 6 and 8 (IL-6, IL-8), and tissue inhibitor of metalloproteinase 1 (TIMP-1) [87,88]. The interaction of OC cells with adipocytes is also regulated by fatty acid binding protein 4 (FABP4), which is an intracellular chaperone for free fatty acids induced during adipocyte differentiation [77], and salt-inducible kinase 2 (SIK2), which is expressed in OC cells [89]. Additionally, research conducted in vitro and using animal models has demonstrated that omental adipocytes facilitate the migration and metastasis of OC cells through the interaction of omental monocyte chemoattractant protein-1 (MCP-1) with its chemokine CCR-2 receptor, expressed on OC cells. This results in the activation of the PI3K/AKT/mTOR pathway and its downstream effectors, HIF-1α (hypoxia-inducible factor 1-alpha) and VEGF-A (vascular endothelial growth factor A) [87].

Molecules that play an important role in the regulation of OC cell metabolism, as well as proliferation, apoptosis, angiogenesis, and migration in the context of cancer metastasis, may be the targets of novel therapies. One such potential target for treatment is the salt-inducible kinase 2 (SIK2), a key element in the interplay between OC and adipocytes found within the OC’s surroundings [90]. Preclinical research on ovarian tumors has demonstrated a high therapeutic efficacy of the already available SIK2 inhibitors. Nevertheless, the practical application of SIK2 inhibitors may be limited due to considerable off-target effects that may arise from direct delivery [91].

The migration and colonization of OC cells to the omentum are supported by omental macrophages. They secrete chemokine ligands that bind to the cancer chemokine receptor 1 (CCR-1) and activate the ERK1/2 and PI3K pathways [88]. The experimental evidence obtained in studies on metastatic OC in a mouse model suggests that it is a unique subset of tumor-associated macrophages (CD163+ Tim4+) that are responsible for the invasive progression of cancer cells [92].

Also, the neutrophil influx into the omentum is a critical step in the metastatic spread of OC. This is facilitated by neutrophil extracellular traps (NETs), which are chromatin webs that bind OC cells and, thus, promote metastasis [93]. In fact, these structures possess a number of protumoral properties. They can activate the epithelial-to-mesenchymal transition (EMT), increasing cell invasiveness, trapping and arresting circulating tumor cells, supporting their proliferation, stimulating the reawakening of dormant cancer cells, inhibiting the immune response, and increasing the resistance to oncological therapies. Furthermore, NET proteinases can degrade the extracellular matrix, promoting cancer cell extravasation [94,95]. Early-stage OC was shown to be characterized by the presence of induced omental neutrophils, which undergo a unique form of cell death called NETosis, leading to NET extrusion. It was demonstrated that neutrophil influx and neutrophil extracellular trap (NET) formation are implicated in providing the premetastatic omental niche that helps with the implantation of OC cells [93].

The survival and metastasis of OC cells is regulated by various cells present in the omentum. Neutrophils, together with fibroblasts, and the above-mentioned macrophages and adipocytes were found to promote cancer progression [96,97,98]. Cancer-associated fibroblasts (CAFs), which are a highly heterogeneous subpopulation of stromal cells, stimulate the proliferation of cancer cells, angiogenesis, lymphangiogenesis, immune cell recruitment, and ECM remodeling, and, through the secretion of cytokines and chemokines, they enhance invasion and metastasis. CAFs also form the core of spheroids that serve as scaffolding, allowing cancer cell aggregation [99]. However, the most predominant immune cells in ascites, which suppress inflammation and protect OC cells from anoikis, supporting their proliferation, are tumor-associated macrophages (TAMs) [100].

Ovarian tumor cells grouped with immune and mesenchymal cells display enhanced adhesion to the mesothelium, which is the predominant metastatic site, and they are more invasive. They have the lowest apoptosis rate and show a higher resistance to chemotherapeutics [101,102,103]. The results of in vitro studies have demonstrated that OC cells can interact with fibroblasts and macrophages, and form heterotypic aggregates in ascites [104]. These aggregates are moved around by the mechanical flow of the peritoneal fluid, and they interact with the omentum and the submesothelial extracellular matrix to form secondary tumors [105]. Thus, immune cells contribute to the formation, survival, and growth of aggregates while they are still floating in the peritoneal fluid [106].

### 4.2. Lymphatic Metastasis

Ovarian carcinoma cells have the highest potential to metastasize through the peritoneal cavity. Distant metastasis to the brain, eyes, central airways, heart, bone, skin, or lymph nodes is less frequent. These sites are usually linked with the hematogenous route and lymphatic invasion (Figure 3) [8,11]. However, those routes of dissemination are poorly understood.

Spreading OC cells gain access to the lymphatic system thanks to the presence of the peritoneal fluid. The invasion process occurs through the main drainage channels, located in the diaphragm, which are responsible for the absorption of fluids in the peritoneal cavity. Cancer cells first invade through the basal membrane into the lymphatic vessels, and while traveling with the lymph, they undergo extravasation and, consequently, may form secondary tumors at the metastatic site [107]. In serous OC, lymphatic spread occurs most often in the para-aortic area, while non-serous tumors are characterized by an equal incidence of pelvic and para-aortic metastases [8,108].

Lymphatic metastasis is driven primarily by increased cancer cell motility associated with actin polymerization, actomyosin contraction, and cell adhesion [109]. It is also promoted by factors such as IL-6, IL-8, and VEGF-C. Interestingly, the increased expression of these factors was demonstrated to be correlated with ovarian tumor progression [110].

Systematic lymphadenectomy allows for an accurate stage assessment of early-stage OC. This procedure also has prognostic value, and according to the International Federation of Gynecology and Obstetrics (FIGO) guidelines, lymph node involvement raises the disease stage. Indeed, the rate of lymph node metastases from early OC varies from 13% to 20%, and in patients with advanced stages of cancer, it increases to more than 50% [111]. Consequently, patients with an advanced disease, peritoneal metastasis, or lesions within lymph nodes have a poor prognosis [112].

OC cells can also pass in the vasculature through the pelvic lymph nodes and the left subclavian vein. This leads to the migration of cancer cells to the blood of OC patients [113,114,115].

### 4.3. Hematogenous Metastasis

The hematogenous spread of OC cells is believed to be rare. It was first demonstrated by Pradeep and co-workers using a murine parabiosis model. Their experiments showed that tumor cells entering the bloodstream of otherwise healthy animals predominantly invaded the peritoneal cavity, and especially the omentum and mesentery [114]. The results of another study, also using a murine model of vascular OC metastasis, suggest that OC cells are driven to the omentum, but only in the presence of the ovaries. An oophorectomy abolishes this unique tropism for the peritoneal cavity (Figure 3) [115].

The mechanisms regulating the hematogenous spread of OC cells are poorly understood. The phenomenon of this route of metastasis is explained on a molecular level by the interaction between the Erb-B2 receptor tyrosine kinase 3 (ERBB3) found in OC cells and its ligand, neuregulin-1, expressed by the omental cells [114]. Yet another study suggests that the pro-invasive character of ovarian tumors is determined by the chemokine receptor type 4 (CXCR4) [116].

Key evidence of the significance of hematogenous metastasis is the presence of cancer cells in the bloodstream known as circulating tumor cells—CTCs. Their presence in the blood of OC patients is well documented [4,114,117,118,119,120,121]. It has been confirmed that CTCs provide useful diagnostic and prognostic information linked to both primary and metastatic ovarian tumors as well as recurrent tumors [122]. Since OC progression from intraperitoneal tumor sites occurs earlier than distant metastases, the detection of CTCs may be associated with an adverse clinical outcome [121,123]. The CTC detection rate is higher for advanced stages of OC. As much as 96% of stage IV tumors are CTC-positive, while for stage I tumors, CTCs were detected in only 50% of cases [124]. Consequently, CTC number alteration during chemotherapy may reflect the response to treatment. Among OC patients, CTCs are correlated with the overall tumor severity, and their status assessed before the start of systemic therapy is linked to clinical outcomes such as a shorter progression-free survival (PFS) and overall survival (OS) [123,125,126]. Indeed, two or more CTCs present in an OC patient’s blood are related to an unfavorable prognosis in relapsed OC [127]. Previously untreated patients with advanced stages of OC and a high CTC number (≥3) before chemotherapy had a significantly shorter PFS compared with patients with <3 CTCs. Similarly, the patients with sustained elevated CTC counts ≥2 at baseline and follow-up had a shorter PFS and OS compared with patients with <2 CTCs [122].

Studies assessing the usefulness of CTCs as a marker of the chemotherapy response have demonstrated that the treatment of OC patients leads to a rapid decline in the CTC counts [122]. The overall CTC number may decrease over time at an average linear rate of 1 cell per 10 months during chemotherapy. Platinum-resistant patients were also characterized by significantly higher CTC numbers compared to platinum-sensitive patients [128,129]. CTCs expressing ERCC1 (excision repair cross-complementation group 1) and cyclophilin 3 were recognized as markers of platinum resistance and a poor prognosis [120,122]. During follow-up studies, the increase in the cell number was more informative for predicting progression or relapse than CA125 [130]. Thus, the results indicate that CTCs may be a predictor of OS. CTCs can be found in all stages of OC. Since blood sampling is less invasive and additionally enables serial measurements, CTC analyses provide a minimally invasive method to make diagnoses and monitor the therapy response, as well as provide information that may lead to a treatment modification, especially in the case of minimal residual disease and disease relapse. CTC detection in the blood of OC patients is based on the cell enrichment methods using epithelial (EpCAM, MUC16, MUC1, KRT7, KRT18, and KRT19), mesenchymal (vimentin, N-cadherin, Snai2, CD117, and CD146), and stem cell (CD44, ALDH1A1, Oct4, and Nanog) markers. An analysis of the molecular profiles of CTCs is of diagnostic importance [4,118].

A growing body of evidence indicates that CTCs may be organized in clusters that are characterized by a 30−50-fold increased metastatic potential, compared to the same number of isolated single CTCs [131]. In the case of OC, clusters of CTCs consisting of up to 30 cells were identified in more than 60% of cases and their presence was correlated with platinum resistance, a shorter progression-free survival, and a shorter time to progression [126,130]. The clustering and collective migration of cancer cells increases their chances of survival and promotes specific changes, such as stemness and drug resistance [126]. In fact, CTCs may share some common traits with cancer stem cells (CSCs) [31,132]. The presence of CSCs in OC was confirmed fifteen years ago [133,134]. The first CSCs were isolated and identified from the ascites of an OC patient. Recently, it has been shown that both normal ovaries and fallopian tubes and ovarian tumors at different stages of tumorigenesis contain various stem cell populations [135,136] The best-known markers of ovarian CSCs include CD44, CD133, CD24, CD117, Nestin, Nanog, and Oct3/4, as well as ALDH1A1 and ABC transporters. Their expression may indicate tumor chemoresistance, a tumor-initiating potential, invasiveness, and a poor prognosis [137].

## 5. Therapeutic Strategies in OC

The main treatment in OC is surgery. In most cases, it involves removing the ovaries, fallopian tubes, uterus, omentum, appendix (in the case of a mucinous type of OC), and all the metastatic foci that are technically able to be extracted [138]. If the surgical removal of the tumor is not possible, neoadjuvant chemotherapy should be administered. After three to six courses of neoadjuvant chemotherapy, surgery should again be taken into consideration [138]. All patients diagnosed in a clinical stage higher than IA-G1 (highly differentiated cancer), according to FIGO, are obligatorily subjected to postoperative chemotherapy. As a result, all cases of disseminated ovarian cancer require first-line chemotherapy, which is a combination of carboplatin and paclitaxel [139]. As a maintenance therapy, PARP inhibitors (olaparyb or niraparib) are applied.

The surgical removal of all macroscopically visible foci and subsequent adjuvant chemotherapy rarely guarantees therapeutic success, as in 90% of cases, the disease recurs despite a previous response to treatment [140]. Then, a second-line therapy, usually platinum-based chemotherapy, is administered. If the time to recurrence was less than six months, OC is typically classified as platinum-resistant, and the patient is treated with doxorubicin, topotecan, gemcitabine, cyclophosphamide, or another type of chemotherapy [141].

The discovery of tumor-associated molecules and their contribution to OC growth and spread has helped identify new therapeutic targets. The high expression of folate receptor alpha (FRα) on the surface of cancer cells and the ability of FRα to transport cytotoxic drugs into cancer cells have led to the development of various therapeutic modalities, including antibodies, antibody–drug conjugates (ADCs), CAR-T, vaccines, small molecules, and folate–drug conjugates [142,143]. Mirvetuximab soravtansine (MIRV), a FRα-targeting ADC, has recently been approved in the United States by the Food and Drug Administration to treat adult patients with platinum-resistant OC, fallopian tube cancer, or primary peritoneal cancer. Other monoclonal antibodies, such as bevacizumab (Avastin), larotrectinib (Vitrakvi), and entrectinib (Rozlytrek), have been successfully used to target tumor-associated antigens, tumor-promoting molecules, and immune checkpoint molecules [144]. A new therapeutic field in OC is immunotherapy based on PD1/PD-L1 inhibitors or anti-CTLA-4. Nevertheless, so far, no immunotherapeutic agents have been approved for recurrent OC treatment or included in any of the available treatment guidelines [145].

Drugs, which are still scarce in the therapeutical repertoire, comprise agents that block cancer cell migration, which limits invasion and metastasis. Agents affecting the cytoskeleton function, especially actin polymerization and contractility, have been defined as the target mechanisms of potential anti-metastatic drugs [146]. The main process regulating actomyosin contractility is the Rho-driven activation of Rho-kinase (ROCK) [147]. The overexpression of Rho/Rock kinases is linked with OC cell dissemination and the efficacy of the cisplatin action. Thus, the use of kinase-specific inhibitors may block cancer cell invasiveness [100,106]. In fact, the inhibition of RhoA, ROCK1, or ROCK2 by fasudil inhibits OC cell invasiveness, induces their apoptosis, and enhances cisplatin-induced growth inhibition [100]. Similarly Y-27632, a selective ROCK inhibitor, was reported to significantly reduce the LPA-induced invasiveness of human OC CAOV-3 and PA-1 cells [17]. Another study showed that inhibitors of Cdc42 (members of the Rho family of GTPases), such as CID2950007 and R-ketorolac, can significantly inhibit the migration of the human ovarian carcinoma cell lines OVCA429 and SKOV3ip. Additionally, R-ketorolac blocked the cell adhesion, migration, and invasion of SKOV3ip and primary patient-derived ovarian cancer cells [148,149]. R-ketorolac was the first inhibitor used in a P0 clinical trial that verified whether the administration of racemic (R,S) ketorolac after OC surgery leads to the peritoneal distribution of R-ketorolac [150]. The results of the study confirmed that R-ketorolac reached sufficient levels in the peritoneal cavity of OC patients to inhibit Rac1 and Cdc42 activity. This may contribute to the observed survival benefit in women treated with ketorolac [150].

Drugs that target the actin cytoskeleton to reduce both cell division and invasiveness can induce distinct alterations in microtubule dynamics. Microfilaments and microtubules share the bulk of the same signaling pathways, including the Rho GTPases and their effectors. Microtubules appear to influence the activity of Rho GTPases, which, in turn, control MT dynamics [151]. It was shown that Cdc42 controls both the microtubule-organizing center and Golgi apparatus reorientation toward the direction of migration, which is fundamental for metastatic dissemination [131]. In fact, ß-tubulin, one of the components of MTs, is the target of the most powerful classes of anticancer drugs, namely the vinca alkaloids and taxanes. Microtubule-interfering chemotherapeutic drugs, such as paclitaxel and docetaxel, play a key role in both the front-line and recurrent treatment of OC [152]. They are well tolerated by patients and, for many years, have been the standard of OC treatment. Recently, because of increasing chemoresistance to taxanes, epothilones (i.e., ixabepilone semi-synthetic second-generation analog of epothilone B) have become of interest. Ixabepilone may retain activity in taxane-treated patients and overcome the mechanisms of taxane resistance [153]. The clinical data strongly support the use of ixabepilone (alone or in combination) in the treatment of platinum-resistant or refractory OC [154].

Increasing evidence indicates that oncogenesis also affects cytoskeletal intermediate filaments. Changes in the expression patterns of the proteins that build and regulate intermediate filaments are associated with cancer development and progression, and in particular, with an increase in cell migration and invasiveness into the surrounding tissues. These proteins include vimentin, which is upregulated in cancer cells undergoing an EMT [155,156], and keratins, which have an altered expression in many cancers [157]. This points to the need to develop intermediate-filament-targeted therapies, but so far, no compounds specifically targeting these elements are available.

Despite numerous studies and ongoing clinical trials testing new drugs, there is still a need for novel therapeutic agents that can be used for the treatment of OC patients. Thus, further research is needed to reveal the molecular mechanisms of ovarian cancer dissemination and set new goals in the process of developing therapeutics and treatment procedures.

## 6. Clinical Implications of Different Metastatic Routes of OC

Approximately 70% of OC cases are diagnosed at a late stage, when the cancer cells have already disseminated [2,158]. The mechanisms behind OC metastasis remain elusive, and thus, the efficient prevention and suppression of the metastatic disease are hard to accomplish. This also results in a lack of effective treatment strategies currently available for patients with an advanced disease.

Multiple dissemination routes of OC cells increase the metastasis rate, which makes the disease advance fast and usually go unnoticed. The treatment protocols are dependent on the histopathological reports describing the tumor mass, but they do not take into consideration the plasticity of the spreading cancer cells. Changes in the surrounding environment drive alterations to cancer cells. During the dissemination process, they may undergo different modes of transition, giving rise to mesenchymal, amoeboid, and redifferentiated epithelial cells. This allows them to escape therapy. Thus, novel drugs aimed specifically at the mechanisms regulating the switch between different invasion modes responsible for effective metastasis are needed.

The common denominator for all the migratory modes of cancer cells is the dynamic changes to the cytoskeleton. Paclitaxel and the vinca alkaloids are chemotherapeutic drugs that are well known for their suppression of microtubule dynamics. They exert an anti-proliferative effect caused by the blocking of mitosis, and thus, they can be used in the therapy of different cancers, such as ovarian, breast, bladder, prostate, and lung cancers [159]. In OC, paclitaxel in combination with carboplatin is accepted as the standard of care for first-line chemotherapy; however, the efficacy of the treatment is often compromised by drug resistance [159].

The successful clinical use of microtubule-binding agents as anticancer drugs gives promise that the same will be possible for microfilament-blocking agents. The migration and invasion of cancer cells relies on the contractility of the actin cytoskeleton and the formation of specific protrusions (such as lamellipodia or invadopodia), which is a process that involves many actin-binding proteins. Specifically targeting these proteins in cancer cells may turn out to be very beneficial for metastatic patients. Actin-dependent cellular processes can be precisely modulated through the pharmacological inhibition of actin nucleation factors, such as CK-636, CK-548, and CDDO (inhibitors of the Arp2/3-NPF-induced actin polymerization), or wiskostatin (WASP inhibitor), 3-(2-amino-5-bromophenyl)-1H-quinoxalin-2-one (inhibitor of formin-mediated actin assembly), and the fascin inhibitor—migrastatin [159].

The combined targeting of microtubules and tropomyosin 3.1 (Tpm3.1)-containing actin filaments was also shown to result in a strong anti-tumor synergy [159]. Tpm3.1 is overexpressed in many cancer cells [159] and anti-Tpm3.1 prototype compounds, such as TR100 and ATM3507, have presented anticancer activity in vitro and in vivo without a negative impact on cardiac structure and function, which is the major side effect of anti-actin drugs [160,161,162].

To better understand actin assembly inhibition with future therapeutic relevance, further studies are required. Still, many reports support the hypothesis that the cytoskeleton is deregulated in cancer, and several cytoskeletal-interacting proteins strongly affect the migratory and metastatic phenotype of cancer cells. Thus, a specific treatment aimed at cytoskeleton assembly factors is a promising concept for the development of antitumor therapies.

## 7. Conclusions

Metastasis and the invasiveness of OC are strongly dependent on the phenotypical and molecular determinants of cancer cells. The dissemination of OC may occur via transcoelomic, hematogeneous, or lymphatic routes, and each pathway is characterized by different regulatory mechanisms. However, dissemination would not be possible if not for the ability of cells to change their phenotype via different transition modes. The EMT and the MAT, among other transition types, are said to be key events that promote the survival and invasiveness of OC cells. Moreover, molecular transitions determine the mobility modes acquired by cells. OC cells may migrate in a mesenchymal or amoeboid state, and may be found in clusters or spread individually.

Still, the mechanisms behind the transitions in OC remain to be elucidated. Deciphering all these phenomena may provide the tools for a successful diagnosis and treatment, including the development of drugs and/or therapies that block cancer cell dissemination.

However, it should be remembered that, because of the heterogeneous nature of OC cancer and cell plasticity, cancer diagnoses and treatments, especially those targeting different tumor-driving molecules, may vary among individuals. Thus, the detection of OC cells and their dissemination, as well as the further monitoring of disease progression, requires a holistic approach.
